# Sex Differences in Intelligence on the WISC: A Meta-Analysis on Children with Specific Learning Disabilities

**DOI:** 10.3390/jintelligence13020018

**Published:** 2025-02-06

**Authors:** Lorenzo Esposito, David Giofrè

**Affiliations:** DISFOR, University of Genoa, Corso Andrea Podestá, 2, 16121 Genova, Italy; david.giofre@unige.it

**Keywords:** intelligence, WISC, sex differences, specific learning disabilities, meta-analysis

## Abstract

Several studies have examined performance on the Wechsler batteries in typically developing children and adolescents. In particular, some studies suggest that cognitive functioning may differ between males and females. In this framework, the present study aims to investigate, through a meta-analytical approach, whether there are sex differences in the profiles emerging from the WISC battery in children with a Specific Learning Disability. For this purpose, a systematic search was conducted, resulting in a final selection of 12 published studies which utilized the WISC and included at least one group of SLD children of similar ages. Scores obtained in each scale and subtest of the battery were then examined according to the CHC/WISC-V classification. A series of mixed-effects models were fitted to meta-analyze the data. The results highlight some differences favoring males, and others advantaging females. On the one hand, males exhibited higher performances in crystallized intelligence, visual processing, and quantitative knowledge. On the other hand, females showed better performance in short-term memory and processing speed. Nevertheless, no differences in fluid reasoning emerged, which probably did not prompt differences in overall intellectual functioning. From a practical and implications point of view, understanding sex-specific differences seems to be of pivotal importance, since it might trigger the development of ad hoc intervention programs in the clinical and educational context.

## 1. Introduction

There is an ongoing debate on sex differences in cognitive functioning, aiming to understand in which specific skills and abilities males and females may differ (e.g., Flores-Mendoza et al. 2013; Hajovsky et al. 2018; Keith et al. 2008). However, in the past years, most studies on this topic have been conducted on standardization samples limited to a single country (Kush and Canivez 2019; Lecerf and Canivez 2018, 2022; Lynn et al. 2005; Strand et al. 2006) and have predominantly involved adults or typically developing populations (Voyer and Voyer 2013; Giofrè et al. 2022a; Keith et al. 2008; Voyer et al. 2017, 2021). These limitations make it challenging to infer whether sex differences might be generalized to the whole population, both typical and atypical.

Many research findings on this topic can be explained by different yet connected theories. Most of them recognize the presence of both biological (nature) factors, such as physical distinctions, evolved traits, and hormonal influences, as well as socio-cultural (nurture) factors, including social- and cultural-role learning, and ingrained stereotypical beliefs (Geary 2020). For instance, prominent theories suggest that cognitive variations between sexes, especially in spatial tasks, likely stem from differences in brain lateralization (Vogel et al. 2003) and hormone levels (Pintzka et al. 2016). Contrastingly, biological theories have faced strong criticism from other influential authors who argue that group differences in IQ likely have an environmental origin (Nisbett et al. 2012; Sternberg 2018). It is worth noting that none of these theories provide conclusive answers, and the question of whether men and women are fundamentally different or similar demands further investigation (Hyde 2014, 2016; Miller and Halpern 2014; Reynolds et al. 2022), also considering that observed IQ differences often depend on the assessment tools used (Bünger et al. 2021).

The Wechsler Intelligence Scale for Children (WISC) is a prominent and widely used tool for assessing the intellectual abilities of children. It is designed to provide a comprehensive assessment of a child’s cognitive abilities (e.g., verbal comprehension, perceptual reasoning). It is commonly used in clinical, educational, and research settings to gather information about a child’s cognitive strengths and weaknesses, as well as to aid in the identification of learning disabilities, giftedness, and other intellectual or developmental challenges (Wechsler 1949). The first version of the WISC published in 1949 comprised twelve subtests and three scores: Verbal IQ, Performance IQ, and Full-Scale IQ. Approximately 25 years after its first version, the WISC was revised (i.e., WISC-R) in 1974, and the age range was widened from 5–15 to 6–16 years. Subtests such as Digit Span, Mazes, and Coding remained the same, while the Picture Completion subtest was renovated (Wechsler 1974). Concerning the earlier versions, such as the WISC-III (Wechsler 1991), it incorporates twelve subtests from the WISC-R and introduces one new optional subtest called Symbol Search. Symbol Search is specifically crafted to gauge the speed of visual-perceptual analysis and processing. The thirteen subtests are categorized into two groups: six belong to the verbal scale, and seven are part of the performance scale, with only ten being mandatory. The WISC-IV (Wechsler 2003) underwent substantial internal structural modifications to align with the evolving needs of clinical practice and advancements in theories and measurement techniques (Prifitera et al. 2008). In this revision, several new conceptual subtests were introduced, while others considered outdated were eliminated. The WISC-IV comprises a total of fifteen subtests, consisting of ten primary and five supplementary ones. The WISC-V (Wechsler 2014) is the most recent version of the WISC and it is widely used intelligence assessment for children (Flanagan and Alfonso 2017). Since it aligned with the CHC model, it now offers a more comprehensive assessment of cognitive abilities, allowing to estimate five primary indices and five additional indices (i.e., *gQ*). Its structure has been comprehensively examined across a variety of samples (Becker et al. 2021; Canivez et al. 2019; Lecerf and Canivez 2018). Although the WISC-V was introduced about a decade ago, it has just recently been released in several countries. Therefore, studies on SLDs employing the WISC-V are still limited, at least in some countries.

The non-theoretical approach of the WISC distinguishes it from intelligence tests that rely on specific theoretical models. This empirical approach enables the WISC to be used with varied groups and circumstances (Flanagan and Alfonso 2017; Flanagan and Kaufman 2004). However, while this element helps with its broad applicability, it may also limit the results’ interpretability within specific theoretical frameworks. Nevertheless, due to the rise of the CHC theory (named after Cattell, Horn, and Carroll) and the introduction of the cross-battery approach, battery results can be compared using the CHC theory (Flanagan et al. 2000; McGrew 2009). Under the CHC approach, tasks can be categorized based on their associations with the respective CHC factors. Numerous studies conducted on WISC scales have indicated that the CHC framework is valuable in elucidating the structure of the Wechsler scales (Bowden 2013; Wilson et al. 2023a). The CHC model classifies cognitive abilities into distinct levels: at the top of the CHC hierarchy is the *g*-factor, several narrow abilities are at the foundational level, and various broad abilities occupy the intermediate level. Examples of these broad abilities include crystallized intelligence (*gC*), fluid intelligence (*gF*), short-term memory (*gSM*), processing speed (*gS*), visual processing (*gV*), and quantitative knowledge (*gQ*), among others (e.g., Flanagan et al. 2013). In addition to the CHC framework, another theoretical perspective is the Verbal-Perceptual-Rotation (VPR) theory, which emphasizes the role of spatial and mental rotation abilities and could be seen as complementary to the CHC model, which divides cognitive abilities into broad and narrow. According to the VPR model, visuospatial abilities could be split into perceptual and mental rotation abilities (Johnson and Bouchard 2007; Major et al. 2012). It is worth mentioning that alternative theories could be also useful in explaining the sex differences in intelligence. If it is true that the CHC model generally showed a good fit to the data in explaining the covariance among the narrow abilities, at the same time, it does not provide valuable information in terms of cognitive processes. Alternative models that focus on the dynamic relationship among the cognitive functions could be useful when studying sex differences (Conway and Kovacs 2015; Engle 2018; Miyake and Shah 1999).

In regard to sex differences line research, previous findings from the WISC-R indicate a counterintuitive trend where males tend to outperform females in verbal abilities, especially on the information subtest (Born and Lynn 1994; Lynn et al. 2005). Recent research involving the WISC-IV with Italian children reflects a parallel trend, demonstrating a male advantage on most verbal tasks (Pezzuti and Orsini 2016). Additionally, other studies suggest that males exhibit better performance than females on the block design and object assembly subtests of the WISC-R (e.g., Lynn et al. 2005). However, evidence concerning memory tasks is more varied, with some researchers identifying a female advantage in certain verbal memory tasks, while others finding no discernible differences (Hirnstein et al. 2023; Roivainen 2011). In terms of processing-speed tasks, a consistent trend emerges where girls tend to outperform boys in coding. This observation holds across various samples and different versions of the WISC, as documented by studies (Giofrè et al. 2022a). The reason behind this consistent advantage is somewhat elusive, but some authors suggest that it may reflect faster processing in writing speed, associated learning, and quicker retrieval from secondary memory (Halpern and LaMay 2000).

The WISC-IV has been extensively used as one of the most prevalent batteries for assessing children with typical development and those with Specific Learning Disorders (Evers et al. 2012; Flanagan and Kaufman 2004). Referring to SLDs, they are considered a heterogeneous group of disorders involving significant difficulties in acquiring and using skills such as expression, reading, writing, reasoning, and calculation. While there is a high likelihood of comorbidity amongst SLDs, deficits in certain areas can also emerge independently (Prifitera et al. 2008). In fact, SLDs can cause deficits in literacy and math skills (Giofrè et al. 2022b; Moll et al. 2014), as well as within literacy components leading to reading and spelling deficits (Moll and Landerl 2009). A potential issue when investigating SLDs is related to sex ratios. Males tend to show spelling deficits more than females, while females are more impaired in math. However, no sex differences have been found for isolated reading problems or the combination of SLDs (Moll et al. 2014). These sex differences can introduce biases thus distorting research findings and leading to ambiguous diagnoses. It is presumed that these disorders may manifest throughout life due to central nervous system dysfunctions and they are intrinsic to the individual (Fletcher and Grigorenko 2017; Hammill 1990). In addition to challenges in academic areas, children with an SLD may exhibit deficits in other cognitive domains such as working memory and processing speed, which are assessed by several intelligence batteries (Cornoldi et al. 2014; Cornoldi and Giofrè 2014). Intelligence assessment has consistently played a crucial role in diagnosing specific learning disorders, and it is routinely incorporated into the diagnostic process for children with an SLD.

Based on the results historically obtained from the use of the WISC-R on children with learning disorders, it had emerged that males achieved better performances in visual processing tests (e.g., Picture Completion, Object Assembly) (Batchelor and Dean 1990; Spafford 1989; Tittemore et al. 1985), arithmetic (Tittemore et al. 1985), and crystallized intelligence (e.g., Information, Similarities) (Tittemore et al. 1987). Nevertheless, no differences emerged in fluid reasoning (Ackerman et al. 1983; Batchelor and Dean 1990; Vance et al. 1980) and *g*-factor (Canning et al. 1980; Schellinger and Beer 1991; Wiesner and Beer 1991). In addition, an advantage favoring females emerged in processing speed (Batchelor and Dean 1990; Spafford 1989; Tittemore et al. 1985). Following this line of research, more recent work highlighted similar results with newer versions of the WISC (e.g., WISC-IV). Specifically, the examined study considered a large sample of children with dyslexia, dyscalculia, and mixed deficits. Focusing on individual subtests, males scored higher in those indicative of VCI (Verbal Comprehension Index), namely the Similarities, Vocabulary, and Comprehension tests, as well as Block Design, while females outperformed males in the remaining six, namely the Picture Concepts, Matrix Reasoning, Digit Span, Letter-Number Sequencing, Coding, and Symbol Search tests. It is important to note, however, that the differences found, although statistically significant, were generally small in magnitude, except for the Coding test, where the difference in favor of girls was substantial (Giofrè et al. 2022b).

In conclusion, research conducted with various versions of the WISC has demonstrated that sex differences in general intelligence, when they exist, are not particularly large and are unlikely to reflect real differences in the g-factor, but rather variations due to individual subtests included in the battery. Several subtests have shown some sex differences, but the Coding test exhibited the largest difference. Given these foundational principles, our objective was to examine sex differences in cognitive abilities as manifested in the indices and subtests of the WISC. Our focus, in particular, was on the Full-Scale Intelligence Quotient (FSIQ) among the indices. Furthermore, we decided to adopt the CHC theory, employing subtests as indicators of the underlying broad factors, since research on WISC batteries found higher reliability scores among indices rather than single subtests (Watkins and Smith 2013). We chose this framework because it is widely used and makes it possible to include several subtests in a unified structure, which increases the statistical power of our analyses.

To accomplish our goal, we employed a meta-analytic approach, gathering all available evidence regarding female/male differences on the WISC. Our objective was to assess the existence of differences in CHC indices and, if present, to quantify these differences. We did not anticipate finding substantial differences in the Full-Scale Intelligence Quotient (FSIQ) or in fluid intelligence. However, we expected differences in certain broad factors: a female advantage in processing speed, and a potential male advantage in visuospatial abilities.

## 2. Materials and Methods

### 2.1. Systematic Search and Screening

We searched the following databases via a query search for published and unpublished literature: PsycINFO, PubMed, and Scopus. The literature reviewed covered journal publications and doctoral theses published between 1949 and May 2024. The extended timeframe was chosen to provide a comprehensive overview of the application of the WISC battery in children with SLD. Search terms used for this meta-analysis were meant to find and include as many studies as possible that included comparisons between sexes in performance at the WISC battery in SLD individuals. The pre-defined search query was: (“Wechsler Intelligence Scale for Children*” OR WISC*) AND (“Gender difference*” OR “Sex difference*”) AND (“specific learning*” OR atypic* OR disabilit*). The wildcard “*” was used where variations could occur and to take into account plurals (e.g., “difference”/“differences”). All search terms were combined in the same way on all databases, and changes in the syntax were made properly. The search terms were chosen to include as many papers as possible about atypical populations. Specifically, rather than using specific diagnosis terms, a larger research query was chosen that encompassed several diagnoses.

**Figure 1:**
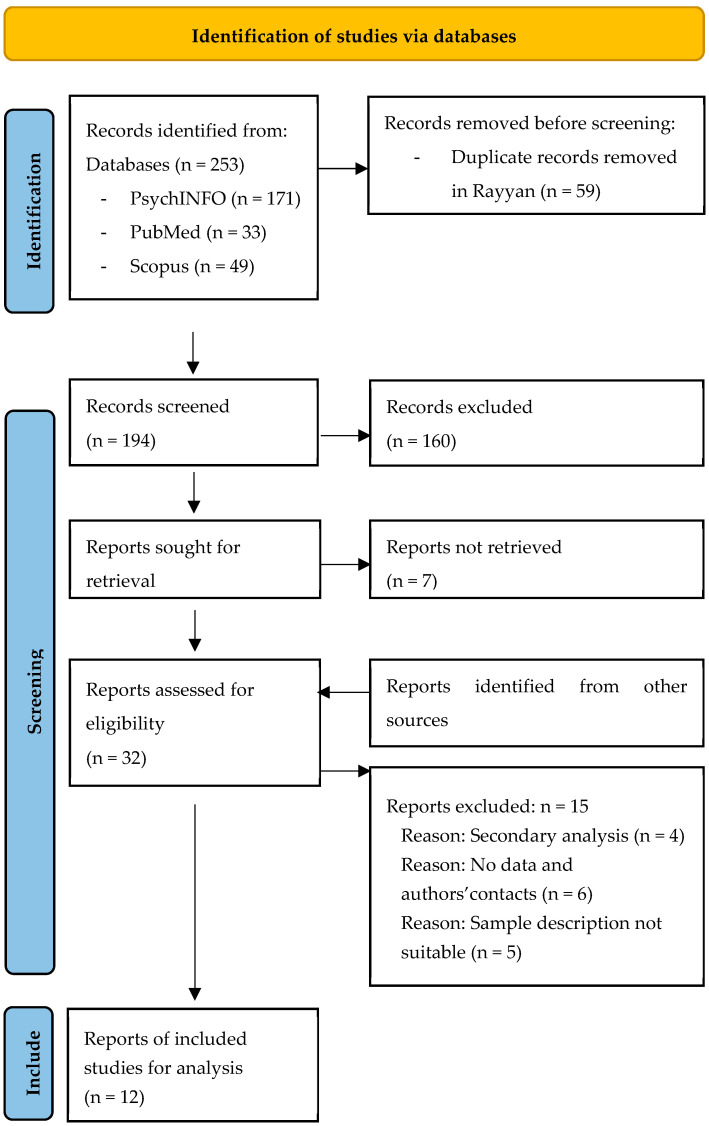
Flowchart of the systematic search and screening.

After conducting systematic searches in each of the three databases, 247 records were collected in total (see Figure 1). Successively, we uploaded the remaining records into Rayyan (Ouzzani et al. 2016). Records have been cleaned of duplicates according to their DOI first, leaving a total of 188 records. The procedure and the reporting style for the current review are in accordance with the Preferred Reporting Items for Systematic Reviews and Meta-Analyses 2020 (PRISMA 2020) statement (Page et al. 2021). Two independent reviewers independently evaluated each article and decided whether each specific paper was to be included in the meta-analysis. Disagreements were resolved by a third reviewer. In the first stage, a rating of the abstract for each record was performed. During this phase, any record that clearly did not involve any version of the WISC, or comprising data collected only on typical samples was excluded.

### 2.2. Data Extraction and Coding

In the second phase, full-text screening has been made, and at that stage, studies satisfying eligibility have been included in the final database. The inclusion criteria were as follows: studies written in English or in any other language the reviewers could understand (e.g., Spanish or Italian); studies reporting primary research data based on at least one index or subtest of the WISC; participant samples that included at least one subgroup of children with atypical development; studies that provided sample size and means for males and females, or at least Cohen’s d or another effect size measure with enough information to estimate its variance (e.g., sample size, standard error) if descriptive statistics were unavailable; participant groups where males and females had a comparable age or belonged to the same age range; and comparisons in which males and females were not matched on any measure of intelligence (e.g., Full-Scale Intelligence Quotient, FSIQ). The latter approach was used since matching participants based on IQ scores could hide real cognitive differences at the score level, leading to biased and unreliable results. If a full-text paper was being considered for exclusion, authors were contacted via email for additional information, and one month was given for them to reply to the email. The two reviewers evaluated all the records. The agreement between the two reviewers was: 95% for the abstract screening phase and 96% for the full-text analysis phase. We conducted a quality assessment to ensure that only studies of sufficient rigor were included. When the findings were not clear, we contacted the authors directly for clarification.

The final database was structured so that each row represented an effect size. Because multiple effect sizes could typically be calculated from each study (for instance, when a single study reported data for several subtests or indices, and possibly multiple samples of children), the number of rows exceeded the number of studies. The database included details about the sample, such as total sample size, participants’ age, nationality, sex proportions, and whether the sample had been used for battery standardization in any country. Wherever possible, this information was coded separately for males and females. For experimental or intervention studies, only data from control groups or pre-test periods were entered.

Information about the measurement instrument, such as the battery version (e.g., WISC-R, WISC-IV) and whether the score reflected an index or a subtest, was also gathered, including the specific name of the index or subtest and, if feasible, the CHC/WISC-V classification (Flanagan et al. 2013; LeAdelle et al. 2005). As for the latter, these levels were coded: *g*-factor (FSIQ scores only), *gF* (subtests: Matrix reasoning, Picture concepts, Picture arrangement), *gV* (subtests: Block design, Mazes, Object assembly, Picture completion), *gC* (subtests: Comprehension, Information, Similarities, Vocabulary), *gSM* (subtests: Digit span, Letter Number Sequencing), *gS* (subtests: Coding, Symbol search), and *gQ* (subtests: Arithmetic). We calculated effect sizes based on subtests unless only index scores were available. The only exception was the Full-Scale Intelligence Quotient (FSIQ), which was always coded if reported, but analyzed separately.

### 2.3. Analytical Approach

#### 2.3.1. Effect Size Computation

Separate effect sizes have been computed for each sample and each measure as Cohen’s *d,* with *d* = (*M*_m_ − *M*_f_)/*S*_w,_ where *M*_m_ is the mean for males, *M*_f_ is the mean for females, and *S*_w_ is the pooled within-sex standard deviation (Borenstein et al. 2009, pp. 21–32). Positive values of *d* represent higher scores for males than females, whereas negative values represent higher scores for females. Where descriptive measures could not be retrieved from full-text screening or directly from authors, statistical estimates have been converted into Cohen’s *d* (Borenstein et al. 2009, pp. 45–49).

#### 2.3.2. Publication Bias

Evaluating publication bias posed challenges for two main reasons. Despite the modest absolute level of heterogeneity, it was substantial relative to the small effect sizes under investigation. The standard deviation (*τ*) among genuine effect sizes often approached or exceeded the magnitude of the effect size (*d*). This high degree of heterogeneity presents a challenge for conventional meta-analytic methods in reliably assessing publication bias (Stanley 2017). To address the aforementioned challenges, the Egger meta-regression approach has been implemented. Egger’s test evaluates the relationship between effect sizes and their standard errors. A significant slope estimate in this model is interpreted as the presence of funnel plot asymmetry, thus indicating the presence of publication bias (Haaf 2021). In addition to the challenges mentioned earlier, it is essential to recognize that asymmetry in the distribution of effect sizes in the funnel plot is not exclusively indicative of publication bias (e.g., Borenstein et al. 2009). Instances where Egger’s test meta-regressions yield estimates larger than the original effect sizes should not be automatically attributed to publication bias; such occurrences are likely driven by heterogeneity alone.

#### 2.3.3. Heterogeneity

Heterogeneity across independent samples was evaluated using *τ*, which estimates the standard error of the true effect sizes, and the *I*^2^ index. The *I*^2^ index indicates the percentage of total variance attributable to the variance in true effects (Higgins et al. 2003). Higher values of *I*^2^ (for instance, *I*^2^ > 75%) suggest that a large proportion of the variance in the observed effect sizes arises from differences in the true effect sizes, implying a notable influence of moderators.

#### 2.3.4. Model Fitting

The RStudio software (R Core Team 2024; Posit Team 2024) was utilized for all analyses. Meta-analytic models were constructed using the multilevel random-effects models function implemented in the “metafor” package (Viechtbauer 2010), following the analytical approach outlined by Borenstein et al. (2009). Random-effects models were employed to accommodate the presumed heterogeneity across effect sizes. To address the dependence structure in the dataset, multilevel models were applied, allowing for the modeling of effects from a structure where multiple samples are nested within studies. Studies and samples have been included as random effects. Multiple effect sizes, when nested within samples, were combined beforehand. The combination of effects was performed using the formulas recommended by Borenstein et al. (2009, pp. 227–29), assuming a correlation of 0.7 among effect sizes within the same sample. Moreover, to maintain simplicity, separate meta-analytic models were fitted for different factors of intelligence.

## 3. Results

A total of 12 studies, published between 1980 and 2024, encompassing 15 distinct independent samples, and 169 effect sizes were included (Table 1). The total number of participants was 2907 (1889 males and 1018 females). Nine studies were conducted in the U.S., one in Canada, one in Italy, and one in Israel (representing approximately 50.472%, 1.668%, 37.541%, and 10.317% of the total sample, respectively). Eleven studies implemented old versions of the WISC (k = 1 for WISC; k = 10 for WISC-R), while the remaining one used a new version (i.e., WISC-IV). Additional analyses were included in the Supplementary Materials (Figures S1–S36). 

### 3.1. Differences in Subtests

A summary of the results by subtest is reported in Table 1. The following results are presented by the classical WISC indices.

Concerning the subtests related to verbal comprehension, all effects were modest in magnitude and in favor of males. The largest difference was observed in the Information subtest, *d* = 0.309, 95%CI [0.203, 0.416], *I*^2^ < 0.001. Following, small effects were found in the Vocabulary and Comprehension subtests, *d* = 0.251, 95%CI [0.111, 0.392], *I*^2^ = 38.049; *d* = 0.305, 95%CI [0.076, 0.533], *I*^2^ = 75.344. As far as the Similarities subtest, again a negligible effect was found, *d* = 0.131, 95%CI [0.030, 0.231], *I*^2^ = 7.009.

Regarding the subtests related to visuospatial processing, all effects were negligible in magnitude and in favor of males. As far as the Block Design subtest, a small effect was found, *d* = 0.288, 95%CI [0.152, 0.423], *I*^2^ = 34.305. Larger differences were found in the Object Assembly and Picture Completion, *d* = 0.407, 95%CI [0.301, 0.514], *I*^2^ < 0.001; *d* = 0.300, 95%CI [0.193, 0.406], *I*^2^ < 0.001. A negligible and non-significant difference was found in the Picture Arrangement, *d* = 0.117, 95%CI [−0.015, 0.250], *I*^2^ = 14.409.

Concerning the subtests related to working memory, mixed results emerged. As far as the Arithmetic subtest, a small difference favoring males was found, *d* = 0.189, 95%CI [0.083, 0.294], *I*^2^ < 0.001. A negligible difference, though not significant, was found favoring females in the Digit Span subtest, *d* = −0.021, 95%CI [−0.113, 0.071], *I*^2^ < 0.001.

Regarding the processing speed, a large difference favoring females was found in the Coding subtest, *d* = −0.420, 95%CI [−0.602, −0.238], *I*^2^ = 60.410.

### 3.2. g-Factor/Full-Scale IQ

Nine studies, encompassing 12 samples, included data for the FSIQ. The effect size was negligible and not significant, with marginally higher scores in females than in males, *d* = −0.051, 95%CI [−0.233, 0.131], *p* = .585. Heterogeneity was fairly small in absolute terms, but sizable when contrasted with the very small effect size, *τ* = 0.245, *I*^2^ = 76.383 (i.e., standard error across true effect sizes is larger than the mean effect size). The Egger method did not suggest any publication bias, as the meta-regression with standard error did not provide significant estimates (b = 0.543, 95%CI [−1.199, 2.287], *p* = .541).

### 3.3. gC/Crystallized Intelligence

Seven studies, encompassing nine samples, included data for crystallized intelligence. The effect size was significant even though negligible, with marginally higher scores in males than in females, *d* = 0.18, 95%CI [0.089, 0.271], *p* < .001. Heterogeneity was again relatively small, *τ* = 0.086, *I*^2^ = 45.386. The Egger method did not suggest any publication bias, as the meta-regression with standard error did not provide significant estimates (b = 0.656, 95%CI [−1.067, 2.379], *p* = .455).

### 3.4. gV/Visual Processing

Seven studies, encompassing nine samples, included data for visual processing. The effect size was significant and modest, with marginally higher scores in males than in females, *d* = 0.272, 95%CI [0.176, 0.367], *p* < .001. Heterogeneity was small, *τ* = 0.081, *I*^2^ = 35.51. The Egger method did not suggest any publication bias, as the meta-regression with standard error did not provide significant estimates (b = 0.109, 95%CI [−1.602, 1.819], *p* = .901).

### 3.5. gF/Fluid Reasoning

Seven studies, encompassing nine samples, included data for fluid reasoning. The effect size was not significant and negligible, with marginally higher scores in females than in males, *d* = −0.038, 95%CI [−0.175, 0.099], *p* < .588. Heterogeneity was relatively modest in absolute terms, *τ* = 0.147, *I*^2^ = 61.885. The Egger method did not suggest any publication bias, as the meta-regression with standard error did not provide significant estimates (*b* = 0.138, 95%CI [−1.634, 1.909], *p* = .879).

### 3.6. gSM/Short-Term Memory

Seven studies, encompassing nine samples, included data for short-term memory. The size was significant and very small, with higher scores in females than in males, *d* = −0.082, 95%CI [−0.152, −0.011], *p* < .05. Heterogeneity was negligible, *τ* < 0.001, *I*^2^ < 0.001. The Egger method did not suggest any publication bias, as the meta-regression with standard error did not provide significant estimates (*b* = 0.049, 95%CI [−1.246, 1.344], *p* = .941).

### 3.7. gS/Processing Speed

Seven studies, encompassing nine samples, included data for the processing speed. The effect size was modest and significant, with marginally higher scores in females than in males, *d* = −0.372, 95%CI [−0.522, −0.221], *p* < .001. Heterogeneity was relatively small, *τ* = 0.169, *I*^2^ = 68.114. The Egger method did not suggest any publication bias, as the meta-regression with standard error did not provide significant estimates (*b* = −0.118, 95%CI [−2.011, 1.775], *p* = .902).

### 3.8. gQ/Quantitative Knowledge

Six studies, encompassing six samples, included data for the quantitative knowledge. The effect size was small and significant, with marginally higher scores in males than in females, *d* = 0.189, 95%CI [0.083, 0.296], *p* < .001. Heterogeneity was small, *τ* < 0.001, *I*^2^ < 0.001. The Egger method did not suggest any publication bias, as the meta-regression with standard error did not provide significant estimates (*b* = −0.36, 95%CI [−1.821, 1.101], *p* = .629).

## 4. Discussion

In recent years, substantial research has examined cognitive differences between females and males. Meta-analytic studies highlighted male advantages in certain cognitive domains such as spatial memory (Voyer et al. 2017), mental rotation (Reilly and Neumann 2013), as well as mathematics (Else-Quest et al. 2010). In contrast, other studies revealed a consistent female advantage in school marks for all course content areas (Voyer and Voyer 2013). Furthermore, sex-related differences have been explored in various facets of human cognition, including reasoning, memory, and intelligence (e.g., Flores-Mendoza et al. 2013; Hajovsky et al. 2018; Keith et al. 2008). Among these, intelligence is a widely researched topic, and studies tried to examine in which specific intelligence abilities males and females could differ (Johnson et al. 2008). The main objective of this meta-analysis was to explore sex differences in intelligence assessed using the WISC. To do so, tasks were categorized based on their belonging to specific CHC broader factors.

In regard to crystallized intelligence (*gC*), a statistically significant difference, albeit small in terms of the effect size, favoring males was found. This result aligns partly with the past literature, in which an advantage for males has been found in typical development children (e.g., Flores-Mendoza et al. 2013). However, the assessment of verbal tasks involves challenges, since factors like anxiety and other personality traits may influence performance (McDermott et al. 2014).

As for the visuospatial processing (*gV*), it was found males outperformed females. These findings are consistent with previous research demonstrating a male advantage in tasks involving visuospatial manipulation of stimuli. Specifically, males tend to outperform females in tasks requiring the generation and mental manipulation of images in memory (e.g., Voyer et al. 2017). It is important to note that SLD is a heterogenous category, including children with reading difficulties, mathematical difficulties, or who have been diagnosed with multiple deficits. The research highlighted that children with specific reading disabilities seem to have impairments in verbal aspects, conversely, children with specific math impairments tend to perform poorer performance on tasks related to visuospatial processing (Toffalini et al. 2017). Therefore, the results mentioned above should be interpreted with caution, also considering the diverse range of specific learning disabilities.

Concerning short-term memory (*gSM*), past studies pinpointed a possible advantage favoring females in SLD children (Giofrè et al. 2023, 2022b), and in typical development children (Giofrè et al. 2022a; Halpern and Wai 2019). For example, a recent meta-analysis showed no sex differences in serial recall and a simple-span task. However, the authors argued that the presentation format could have moderated these results, making any interpretation inconclusive (Voyer et al. 2021). In the present meta-analysis, this difference, albeit minimal, resulted as statistically significant unlike in previous works.

As for the processing speed (*gS*), the results obtained are in accordance with previous findings. Notably, the literature suggests that males consistently scored lower than females across various school cohorts on tasks related to processing speed. One plausible explanation could be that males exhibit poorer performance compared to females due to challenges in sustaining attention and concentration during prolonged engagement in simple and repetitive tasks (Flanagan et al. 2000; Giofrè et al. 2022b).

Sex differences found in quantitative knowledge (*gQ*) resulted statistically significant, although it was small. It is important to highlight that this area is composed exclusively of arithmetic and the latter represents a somewhat mixed ability and an interplay of various cognitive abilities (i.e., working memory).

Regarding fluid reasoning (*gF*), the results show a small and not statistically significant difference favoring females. This suggests that if differences in fluid-intelligence tasks between females and males exist in the population, they are likely to be negligible. Similarly, the present work highlighted a negligible, albeit not significant, difference in FSIQ favoring females. These findings are in contrast with the pattern observed in typical development children (Giofrè et al. 2022a). It is noteworthy that *gF* and FSIQ converge toward the same direction, probably reflecting the fact that *gF* is the most related factor to general intelligence, and this relationship seems to hold across typical and atypical development (e.g., Giofrè et al. 2019; Weiss et al. 2013). Notably, the indices of heterogeneity for *gF* and FSIQ were moderately high, suggesting that a considerable amount of variance was due to sampling variability. It is plausible that these indices involved studies with small sample sizes. However, the residual heterogeneity was considerably high when compared directly with the effect size, suggesting that moderators might account for a portion of residual heterogeneity.

Overall, our findings partly align with those from past investigations carried out on the Wechsler Intelligence Scale for Children. Research consistently shows male advantages in visuospatial and quantitative abilities (Else-Quest et al. 2010; Voyer et al. 2017), whereas females consistently outperform males in processing speed (Giofrè et al. 2022a). In addition, the female advantage in short-term memory represents a novel and unusual finding that should be interpreted with caution and in the context of sex differences in language development and task-dependent features.

Nonetheless, these results should be considered in light of some limitations that could be addressed in successive investigations. First, the number of studies was limited, and in addition, most of them reported collapsed data over sex, making it difficult to carry on comprehensive meta-analyses. Second, the present meta-analysis did not include any study which used the WISC-V. This last version is not available in several countries. This could introduce a bias, as using only older versions of the WISC may limit the generalizability and comparability of the results. Therefore, it would be interesting to repeat the meta-analysis when a consistent amount of research is available. Third, it is critical to recognize that changes made to subtests throughout time can impact the interpretation and comparability of results. Significant changes to items within subtests, even if the overall subtest structure remained approximately the same, may cause variability in measuring certain cognitive abilities. This calls into doubt the consistency of WISC scores across different versions. Future studies should investigate how these changes affect conclusions drawn from data gathered over time.

Specific learning disabilities represent an umbrella term for a variety of similarities, each linked with slightly different patterns of intellectual functioning which may not always emerge during clinical assessment. In fact, impairments of various types often co-occur in clinical settings (Toffalini et al. 2017). To further complicate the picture, it is important to consider that the same term (e.g., in clinical or other research settings) can take on different meanings, and sometimes be even misleading, depending on the context in which it is used (Elliott 2020). Notably, most children with a diagnosis of SLD tend to have a mixed profile, with the co-occurrence of several diagnoses within the SLD spectrum (Toffalini et al. 2017). As a result, differentiating between different SLD subtypes as distinct entities is problematic. This represents a crucial limitation when trying to understand cognitive aspects in atypical developmental subgroups. In the present study, we decided to consider SLD children under a common umbrella term, on the one hand, this last strategy allowed us to increase our sample size, despite the limited number of studies, and overcome issues related to terminology and context. However, this decision made it impossible to perform analysis at the subtypes level, which would have been interesting from a scientific point of view.

In this meta-analysis we did not perform subgroup analyses based on age or WISC versions, which could have proved helpful. The prevalence of older WISC versions may account for some of the findings. These versions emphasized verbal and visuospatial abilities leading to an overrepresentation of certain cognitive abilities, potentially influencing the results. Likewise, the WISC-IV and the WISC-V included additional subtests emphasizing fluid intelligence and processing speed. Although age and WISC version may, at least in part, account for sex differences, the studies included in this meta-analysis did not allow for a comprehensive examination of these factors. Future research should fill these gaps by conducting subgroup analyses focusing on age, and SLD subtypes. This would provide a more comprehensive picture of sex differences over time and across different SLD subtypes.

While the CHC model is widely acknowledged and employed in intelligence theory, it is not without criticism and limits. In fact, some investigations on the WISC claimed that the overlap between certain conceptions, such as fluid intelligence and general intelligence, makes it impossible to draw clear distinctions between these cognitive abilities (Canivez and Kush 2013). Others pointed out that the CHC framework poorly captures the cognitive processing and the relationships among the factors (Conway and Kovacs 2015; Major et al. 2012). It is also worth noting that the use of the CHC model has been questioned when examining sex differences. For example, Johnson and Bouchard (2007) used the VPR model to investigate sex differences in intelligence. Their results indicated that differences were particularly pronounced in the rotation and spatial abilities, which aligns with our findings. Indeed, most tests requiring spatial manipulation of stimuli (e.g., Block Design or Object Assembly) tend to favor males. Again, our findings also align with previous research showing that girls, independently from the battery being used, exhibit an advantage in all speed tasks such as Coding or Perceptual Speed (Giofrè et al. 2024; Johnson and Bouchard 2007). This finding is compatible with the VPR model, in which an evident advantage for females was observed (Johnson and Bouchard 2007). Notably, in the same study, when authors examined the residual ability scores, they found a large sex difference in memory ability, favoring females. This last result is in line with what was observed in the present study. Taken together, these findings suggest that the CHC model may present some limitations when addressing sex differences, while alternative models, such as the VPR, could provide additional valuable insights.

It can be argued that our findings align with socio-cultural frameworks. A male advantage was found in crystallized intelligence, as well as visuospatial and quantitative reasoning. Expectancy-value theory suggests that expectations of success and the value associated with a task might influence behavior (Eccles 1983). In other words, both males and girls’ cognitive task performance may be influenced by their beliefs. Furthermore, parents’ attitudes can influence how much effort males and females put into specific tasks, such as those that require visuospatial and quantitative reasoning for males and those that require verbal abilities for females. These interpretations are particularly relevant in the context of cross-national studies. For example, a recent systematic study found that the CHC model was consistent across distinct subpopulations, regardless of sample characteristics (Wilson et al. 2023b). Similarly, other studies have found strong evidence of intelligence invariance across a variety of countries, although most of them were Western countries (van de Vijver et al. 2019; Wilson et al. 2023a).

In conclusion, our results showed a pattern of results that has not been observed in typical development children. Specifically, the results may suggest a small advantage in *gF* and *gSM* favoring females within the general SLD population. Consequently, it is also reasonable to consider that the intelligence models proposed in the past might be influenced by the population being investigated, with differences emerging specifically in children with neurodevelopmental disorders. Direct implications for the clinical context are that the assessment of the g-factor may stem from variations associated with the specific subtests included in the battery assessment. Clinicians should be careful when selecting subtests since it is critical to choose those that best represent the strengths and weaknesses of children with SLD; also, it is worth noting that some subtests may focus on specific abilities. By selecting subtests that are more appropriate for the individual’s specific cognitive demands, clinicians can acquire a clearer and more accurate picture of their abilities, allowing them to direct more effective interventions.

## Figures and Tables

**Table 1:** Summary of the main meta-analytic results of sex differences by CHC factor.

Subtest/Area	*n* of Studies	*n* of Samples	*n* of Effects	*n* of Males	*n* of Females	Age *M* (in Months)	Age *SD* (in Months)	*d*	95%CI	*p*	*τ*	*SE*
Subtest												
VC (*gC*)	7	9	10	1894	1007	127.076	24.788	0.251	[0.111, 0.392]	<.001	0.109	0.072
SI (*gC*)	7	9	10	1894	1007	127.076	24.788	0.131	[0.030, 0.231]	.011	0.038	0.051
CO (*gC*)	7	9	10	1894	1007	127.076	24.788	0.305	[0.076, 0.533]	.009	0.244	0.116
IN (*gC*)	6	6	7	1195	470	120.388	21.462	0.309	[0.203, 0.416]	<.001	0.000	0.054
BD (*gV*)	7	9	10	1894	1007	127.076	24.788	0.288	[0.152, 0.423]	<.001	0.101	0.069
OA (*gV*)	6	6	7	1195	470	120.388	21.462	0.407	[0.301, 0.514]	<.001	0.000	0.054
PC (*gV*)	6	6	7	1195	470	120.388	21.462	0.300	[0.193, 0.406]	<.001	0.000	0.054
PA (*gF*)	6	6	7	1195	470	120.388	21.462	0.117	[−0.015, 0.250]	.083	0.066	0.068
AR (*gQ*)	6	6	7	1195	470	120.388	21.462	0.189	[0.083, 0.294]	<.001	0.000	0.054
DS (*gSM*)	7	9	10	1894	1007	127.076	24.788	−0.021	[−0.113, 0.071]	.651	0.000	0.047
CD (*gS*)	7	9	10	1894	1007	127.076	24.788	−0.420	[−0.602, −0.238]	<.001	0.174	0.093
CHC Area												
*g*/FSIQ	9	12	13	1868	1176	126.937	23.997	−0.051	[−0.233, 0.131]	.585	0.245	0.093
*gC*	7	9	37	1894	1007	127.076	24.788	0.180	[0.089, 0.271]	<.001	0.086	0.046
*gV*	7	9	25	1894	1007	127.076	24.788	0.272	[0.176, 0.367]	<.001	0.081	0.049
*gF*	7	9	13	1894	1007	127.076	24.788	−0.038	[−0.175, 0.099]	.588	0.147	0.070
*gSM*	7	9	13	1894	1007	127.076	24.788	−0.082	[−0.152, −0.011]	.023	0.000	0.036
*gS*	7	9	13	1894	1007	127.076	24.788	−0.372	[−0.522, −0.221]	<.001	0.169	0.077
*gQ*	6	6	7	1195	470	120.388	21.462	0.189	[0.083, 0.294]	<.001	0.000	0.054

*Note.* *gC*, crystallized intelligence; *gV*, visual processing; *gF*, fluid intelligence; *gSM*, short-term memory; *gS*, processing speed; *gQ*, quantitative knowledge; VC, vocabulary; SI, similarities; CO, comprehension; IN, information; BD, block design; OA, object assembly; PC, picture completion; PA, picture arrangement; AR, arithmetic; DS, digit span; CD, coding.

## Data Availability

Dataset available on request from the authors.

## Supplementary Materials

The following supporting information can be downloaded at: https://www.mdpi.com/article/10.3390/jintelligence13020018/s1, Figures S1–S36.
