# Supplementary material for: Sex Differences in Intelligence on the WISC: A Meta-Analysis on Children with Specific Learning Disabilities
Source: J Intell. 2025 Feb 6;13(2):18. doi: 10.3390/jintelligence13020018 (PMC11856952; doi:10.3390/jintelligence13020018)

Supplementary materials

Sex Differences in Intelligence on the WISC: A Meta-Analysis on Children with Specific Learning Disabilities

Figure S1. Funnel plot for the *g*/FSIQ area

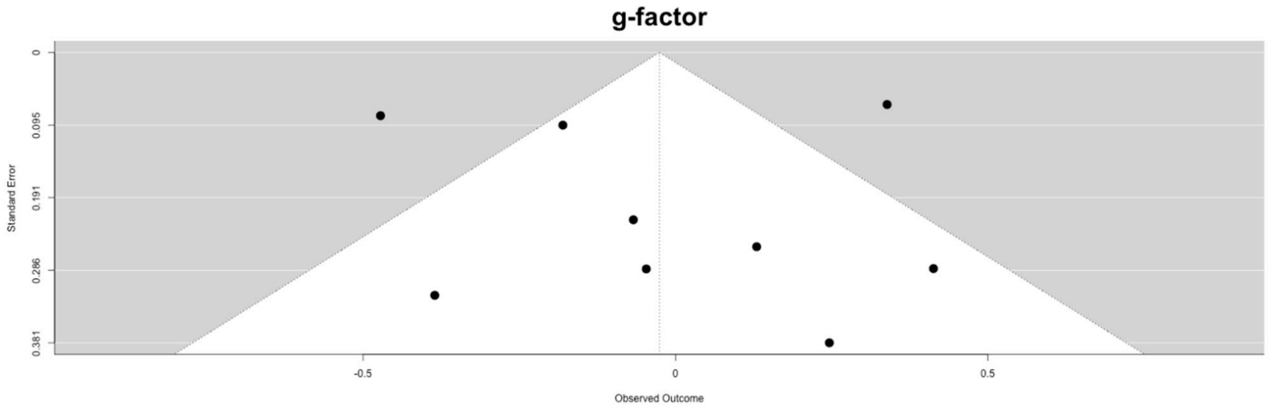

Figure S2. Funnel plot for the *gC* area

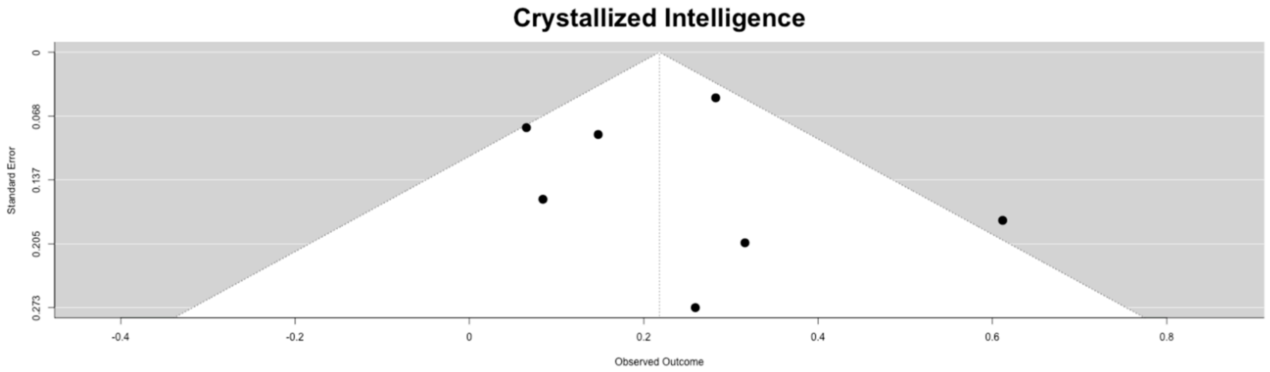

Figure S3. Funnel plot for the *gV* area

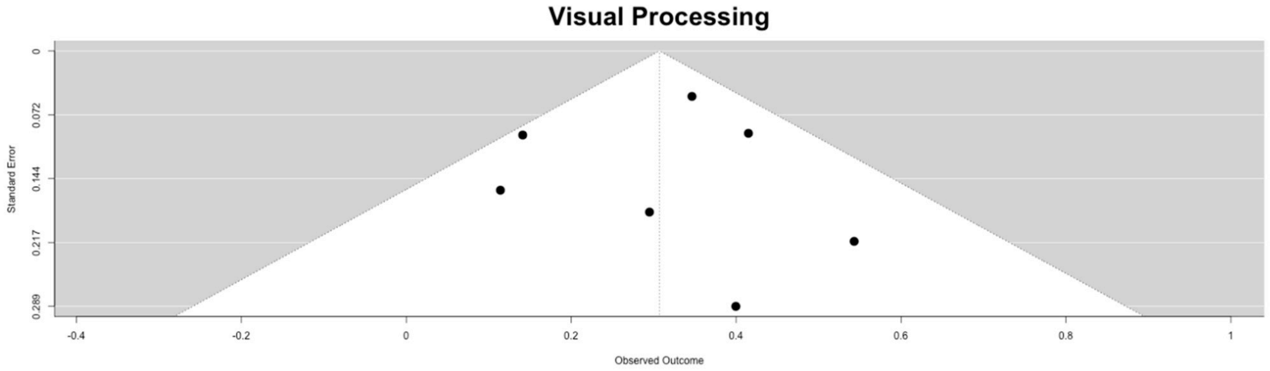

Figure S4. Funnel plot for the *gF* area

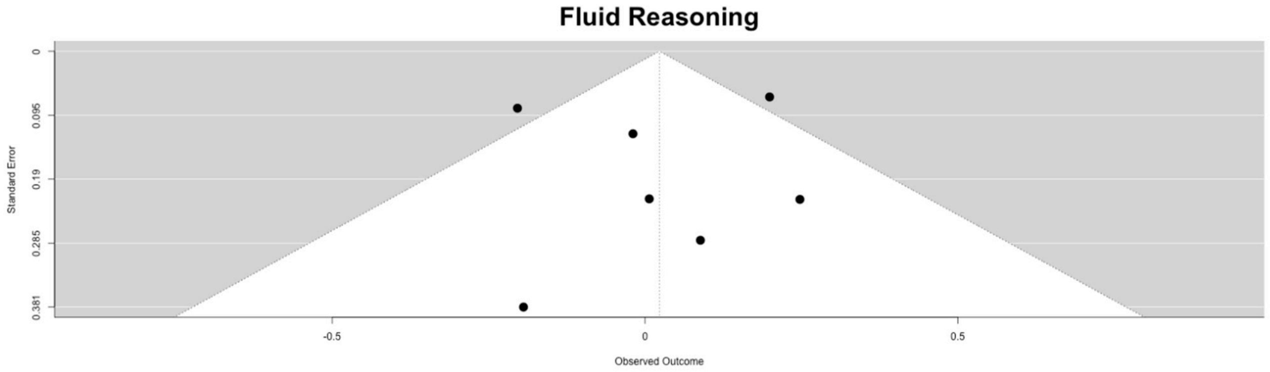

Figure S5. Funnel plot for the *gSM* area

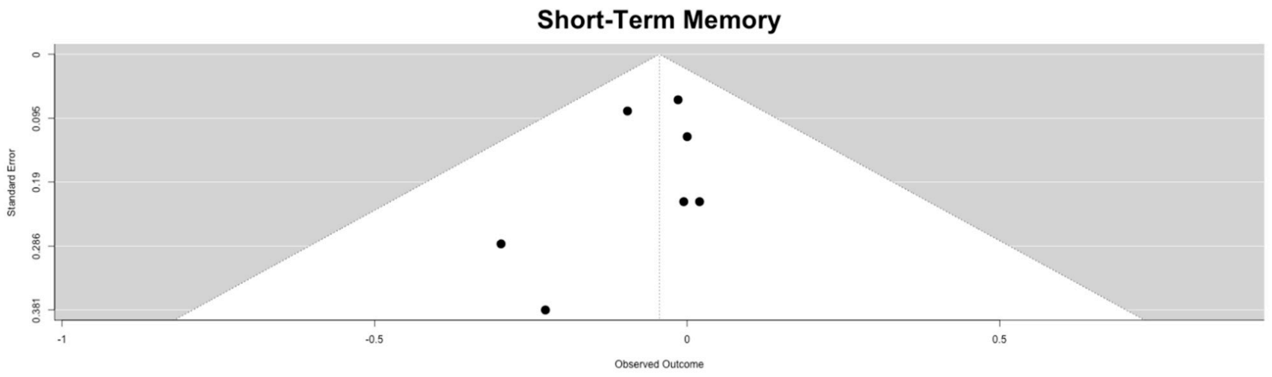

Figure S6. Funnel plot for the *gS* area

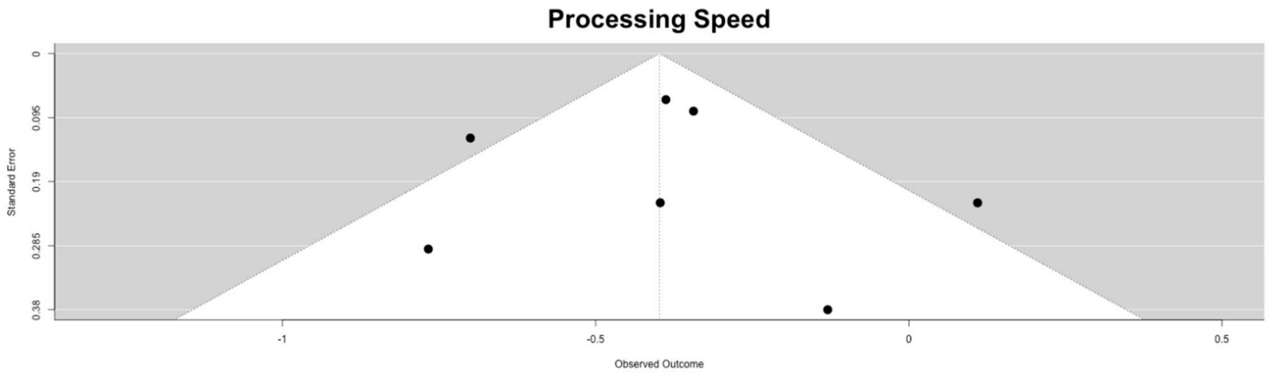

Figure S7. Funnel plot for the *gQ* area

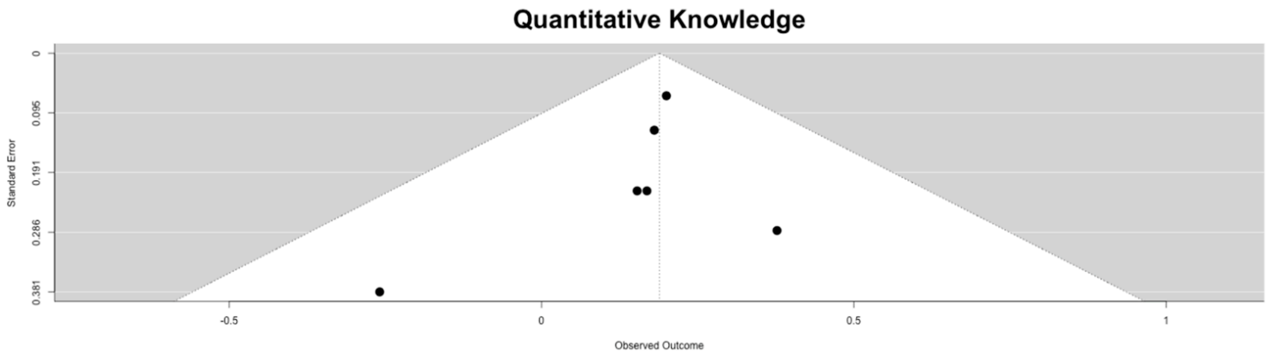

Figure S8. Forest plot for the *g*/FSIQ area

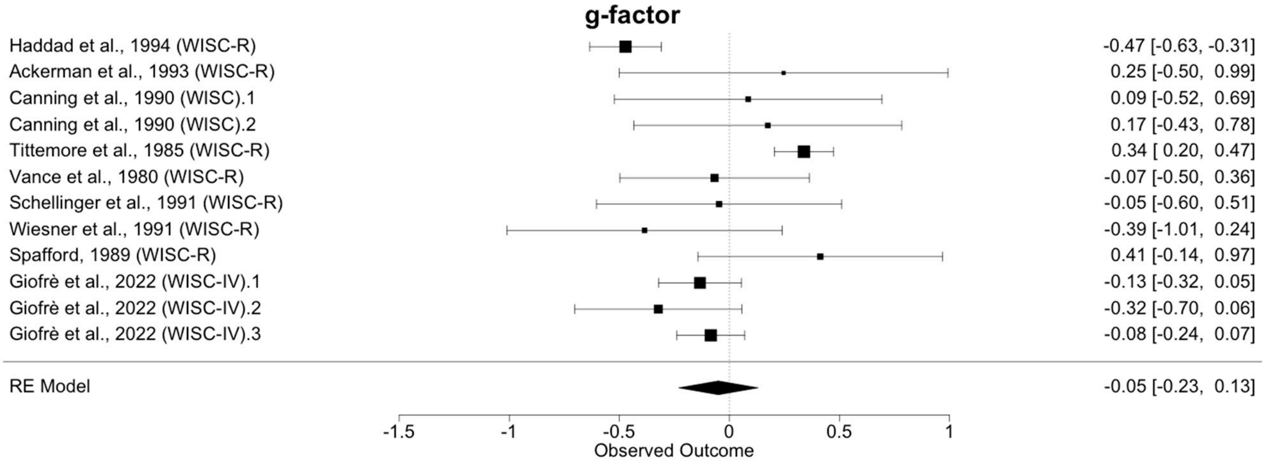

Figure S9. Forest plot for the *gC* area

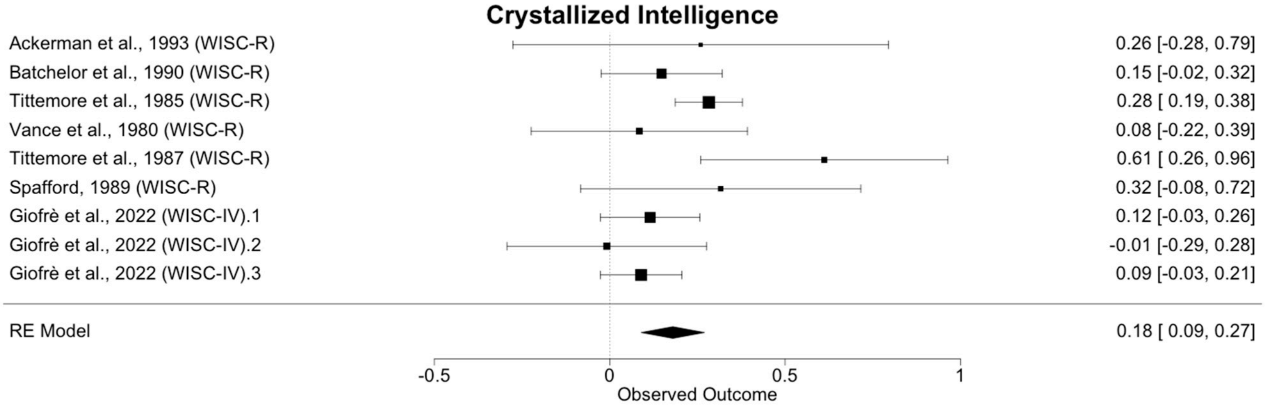

Figure S10. Forest plot for the *gV* area

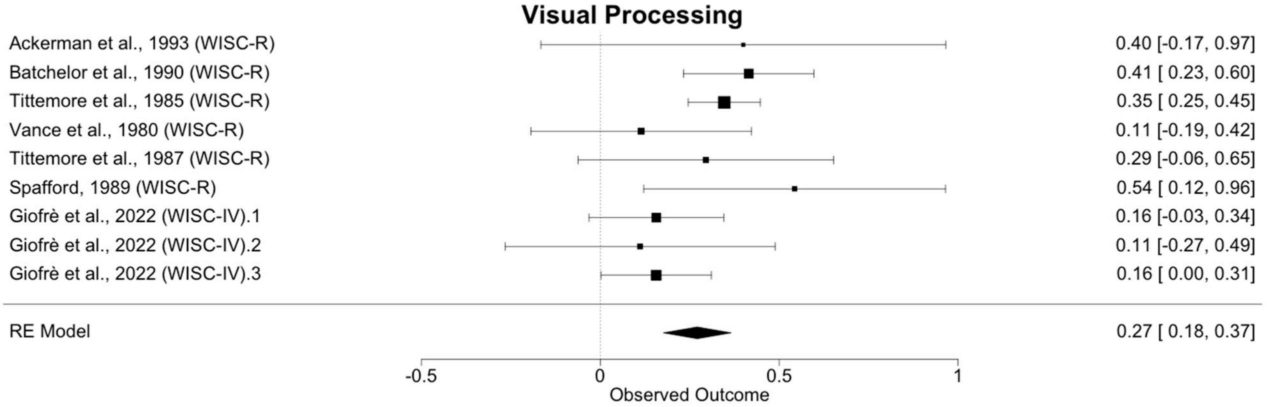

Figure S11. Forest plot for the *gF* area

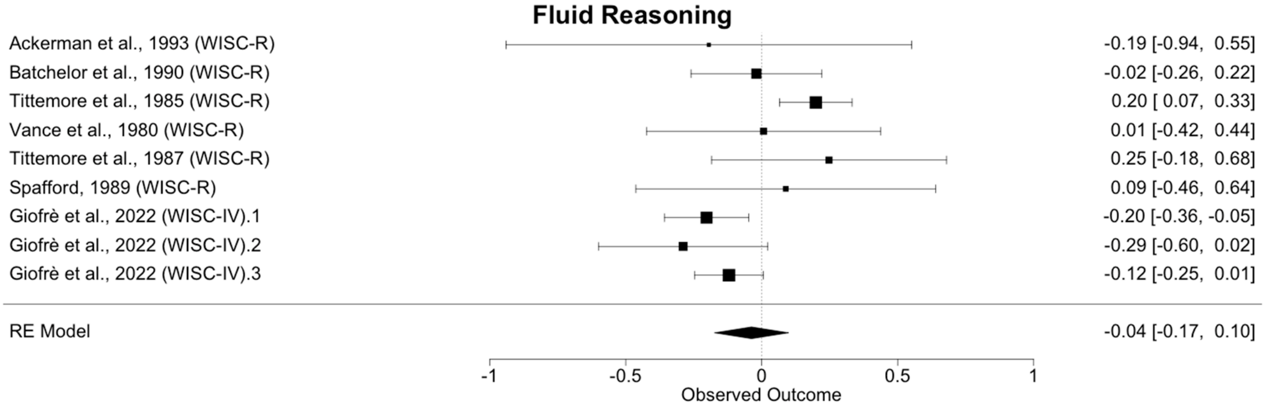

Figure S12. Forest plot for the *gSM* area

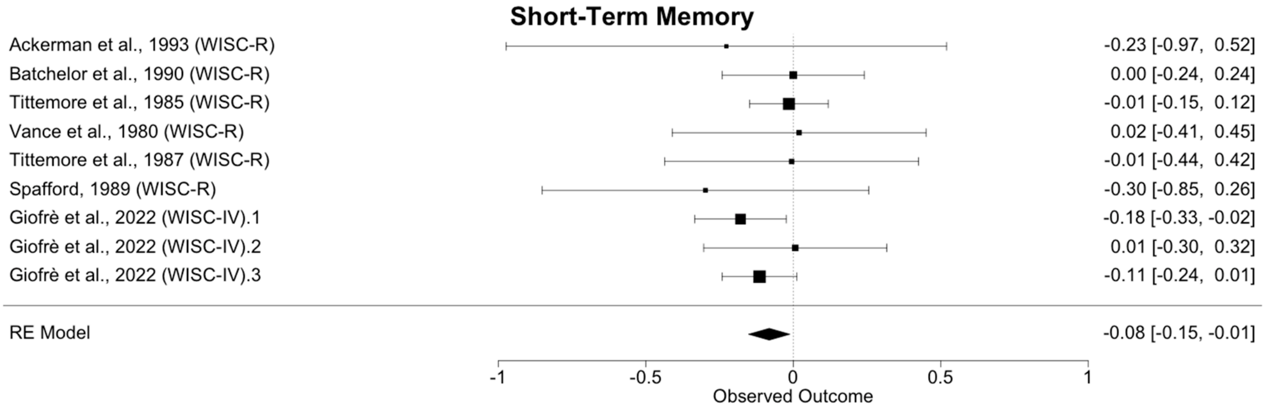

Figure S13. Forest plot for the *gS* area

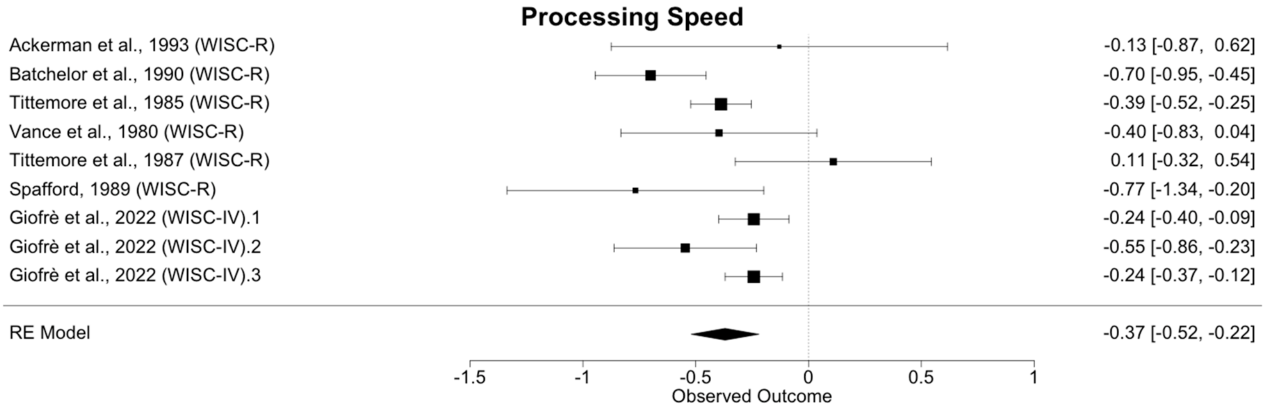

Figure S14. Forest plot for the *gQ* area

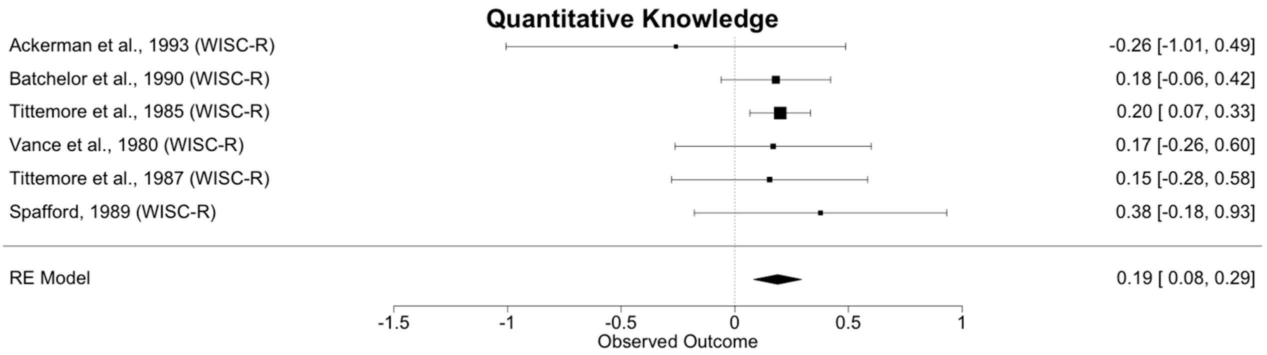

Figure S15. Forest plot for the Similarities subtest

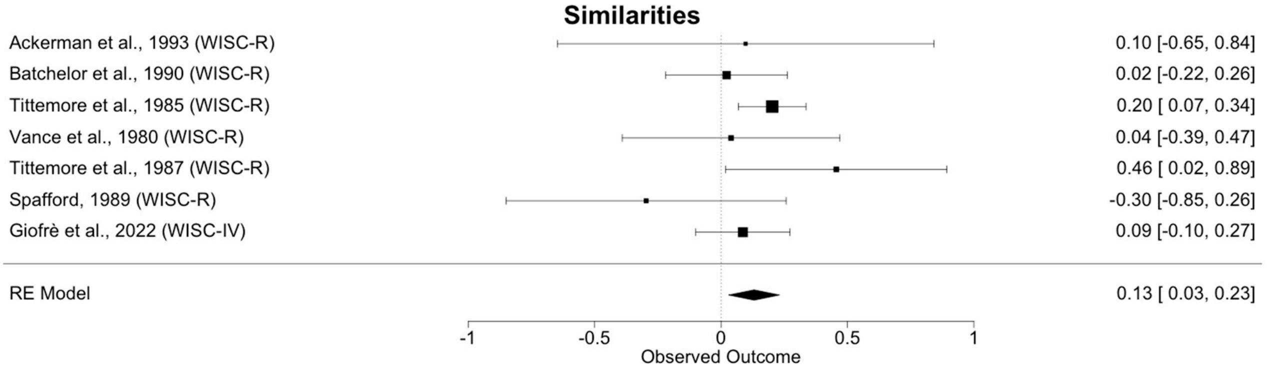

Figure S16. Forest plot for the Coding subtest

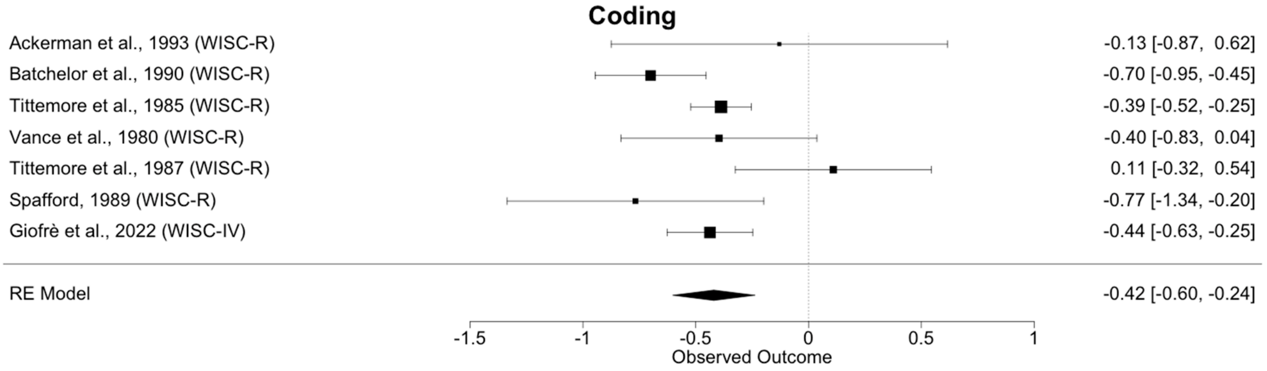

Figure S17. Forest plot for the Block Design subtest

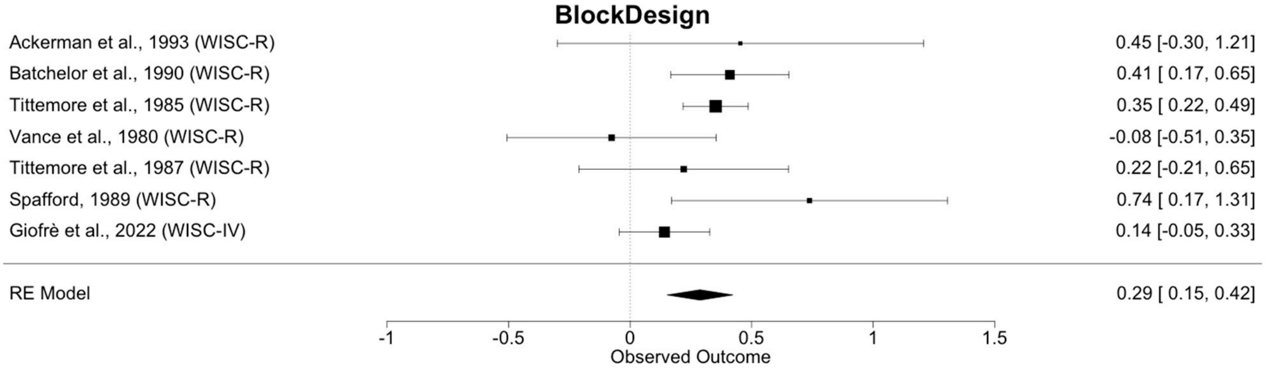

Figure S18. Forest plot for the Comprehension subtest

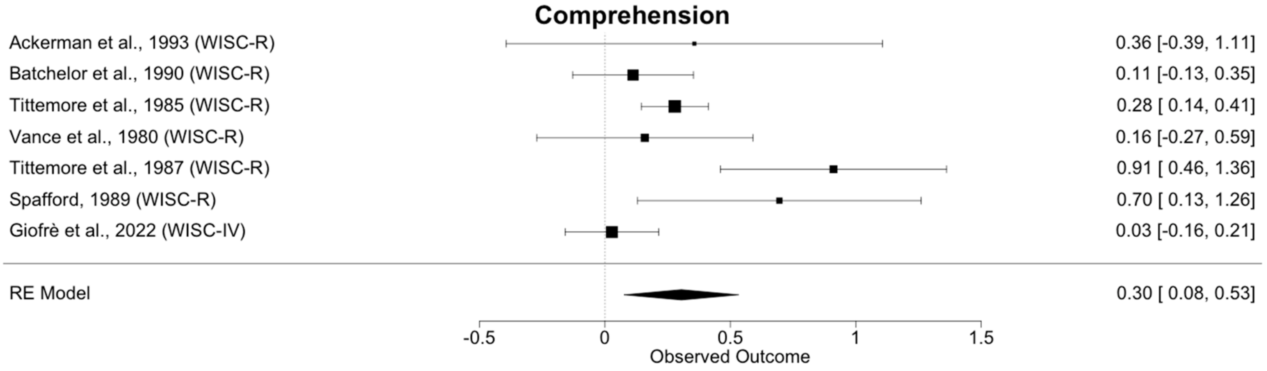

Figure S19. Forest plot for the Digit Span subtest

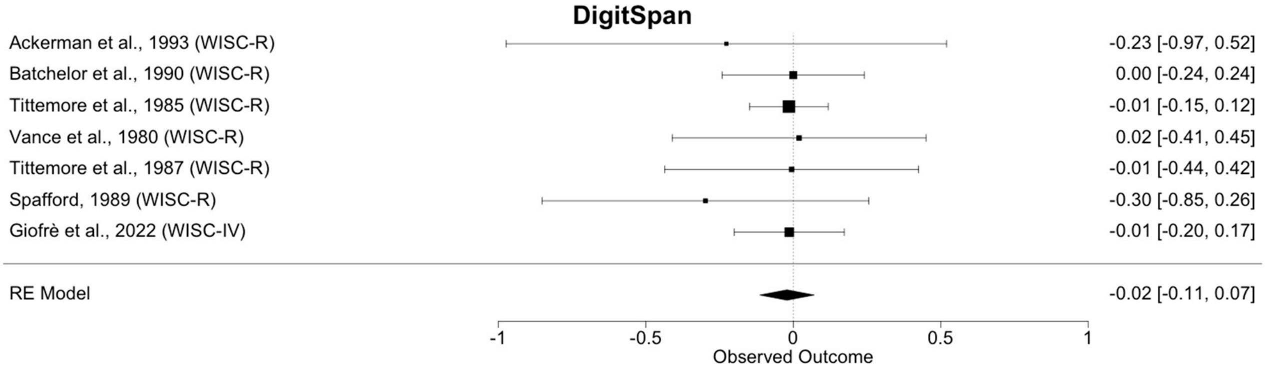

Figure S20. Forest plot for the Picture Completion subtest

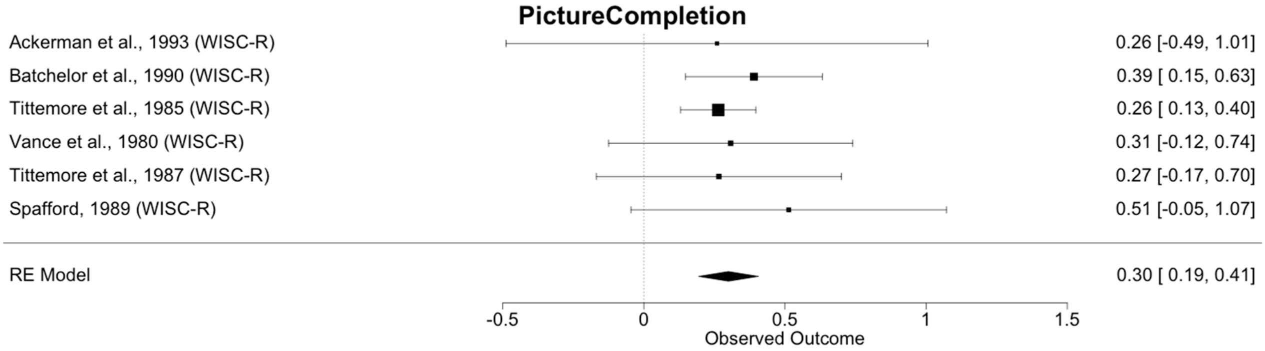

Figure S21. Forest plot for the Object Assembly subtest

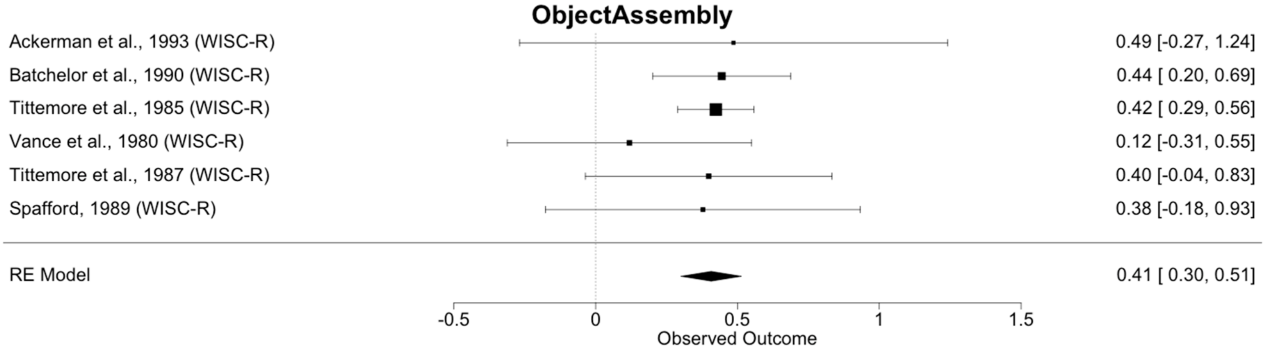

Figure S22. Forest plot for the Picture Arrangement subtest

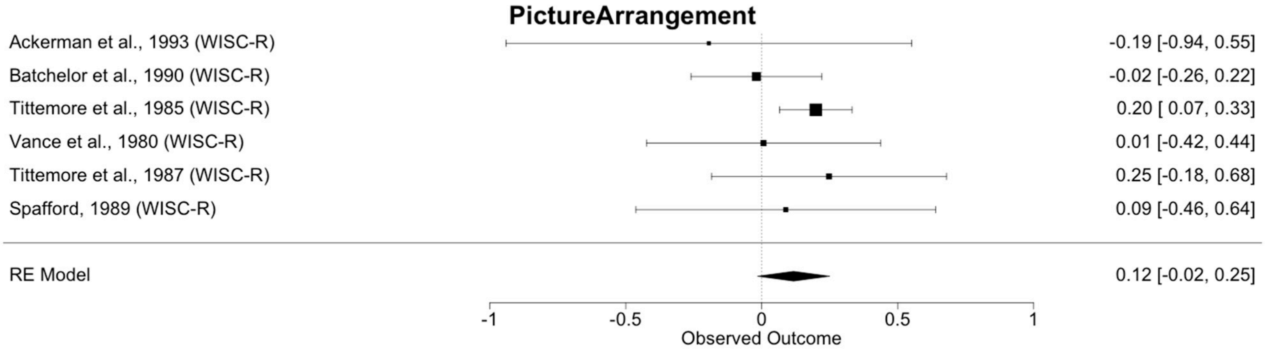

Figure S23. Forest plot for the Information subtest

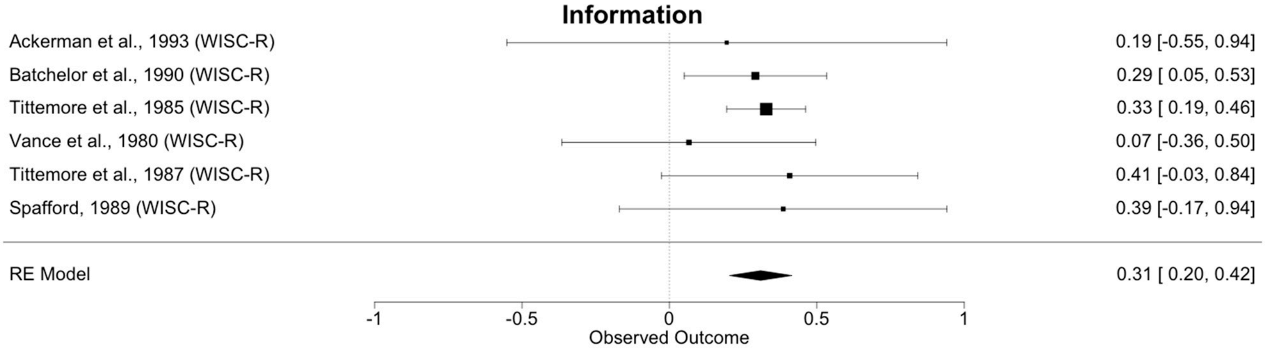

Figure S24. Forest plot for the Arithmetic subtest

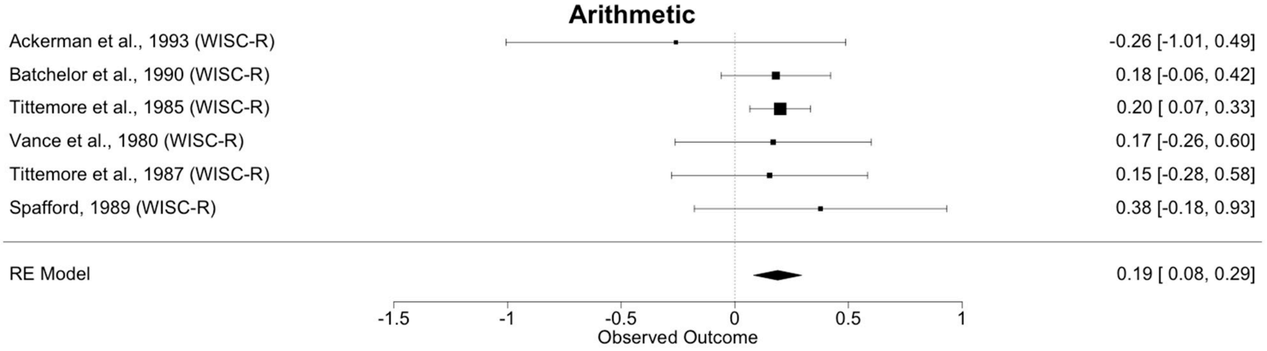

Figure S25. Forest plot for the Vocabulary subtest

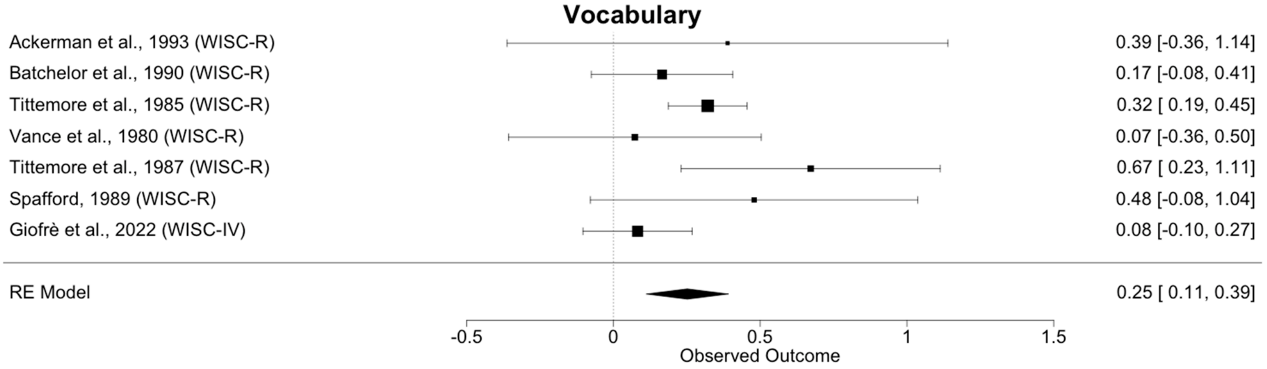

Figure S26. Funnel plot for the Block Design subtest

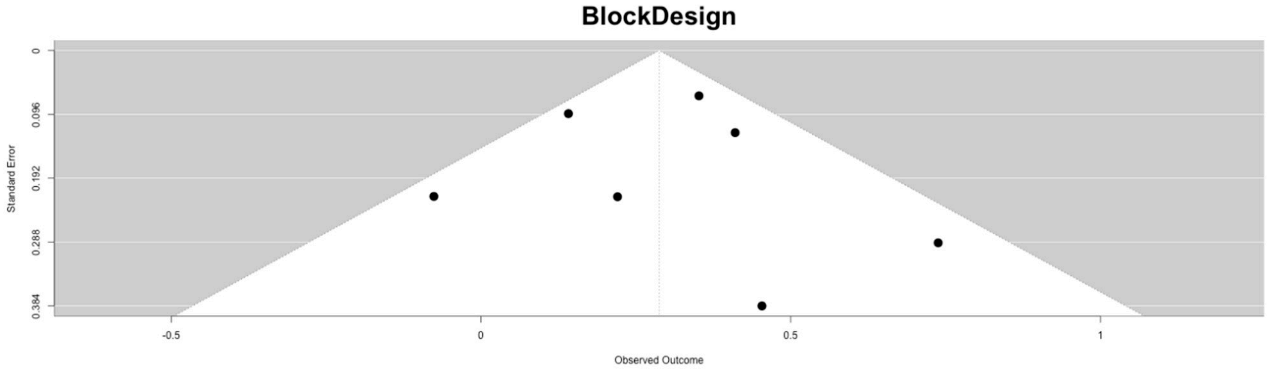

Figure S27. Funnel plot for the Vocabulary subtest

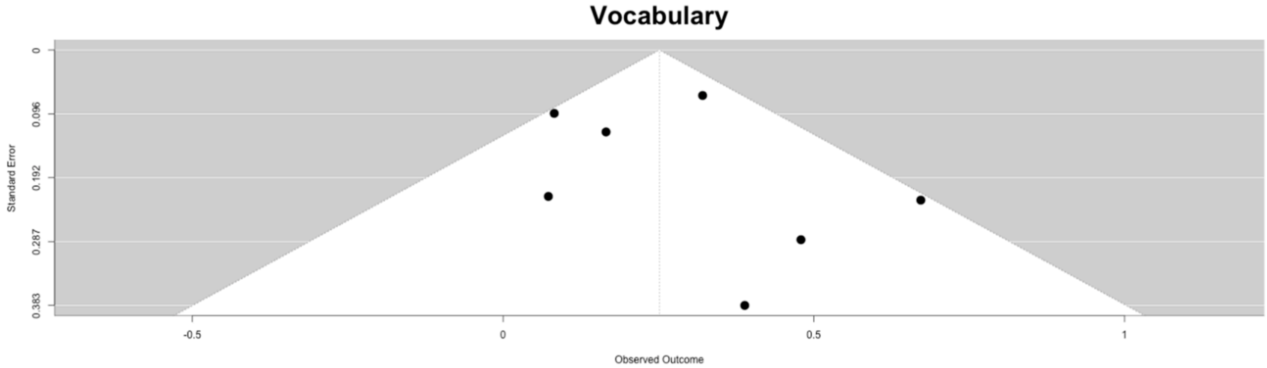

Figure S28. Funnel plot for the Comprehension subtest

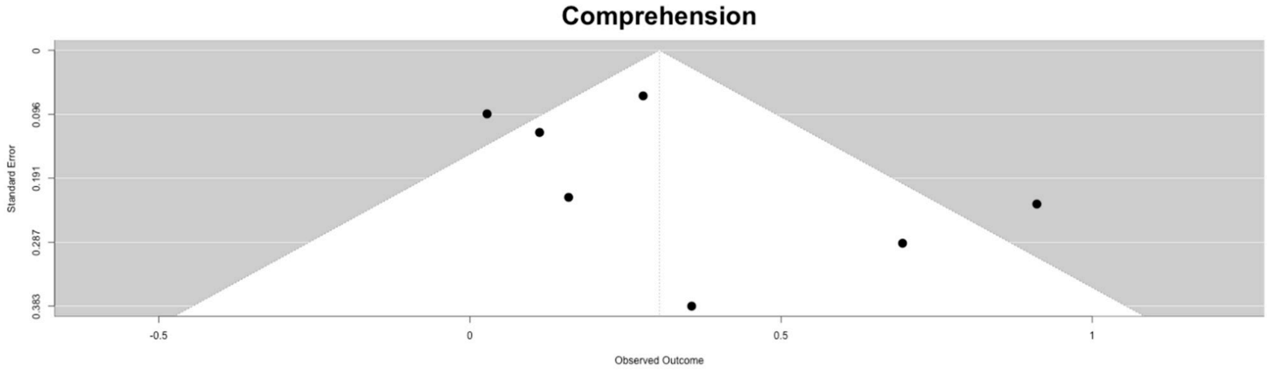

Figure S29. Funnel plot for the Digit Span subtest

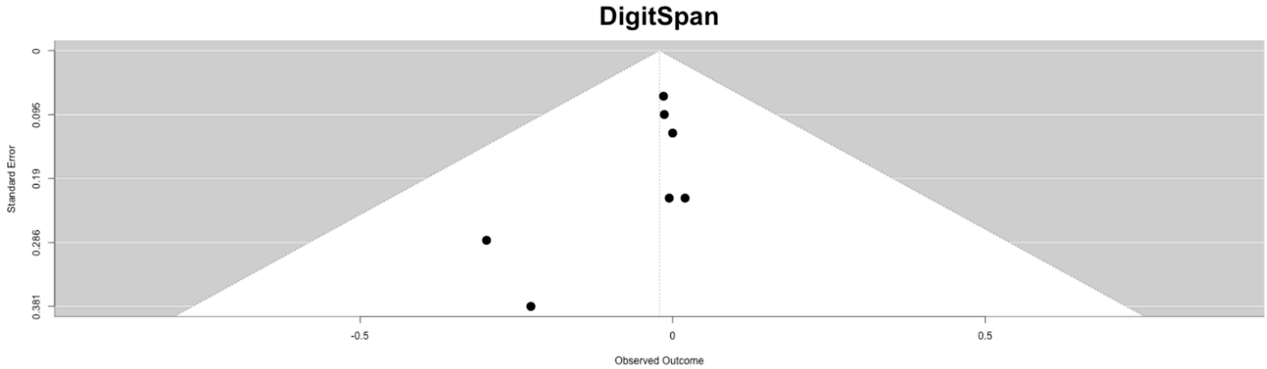

Figure S30. Funnel plot for the Similarities subtest

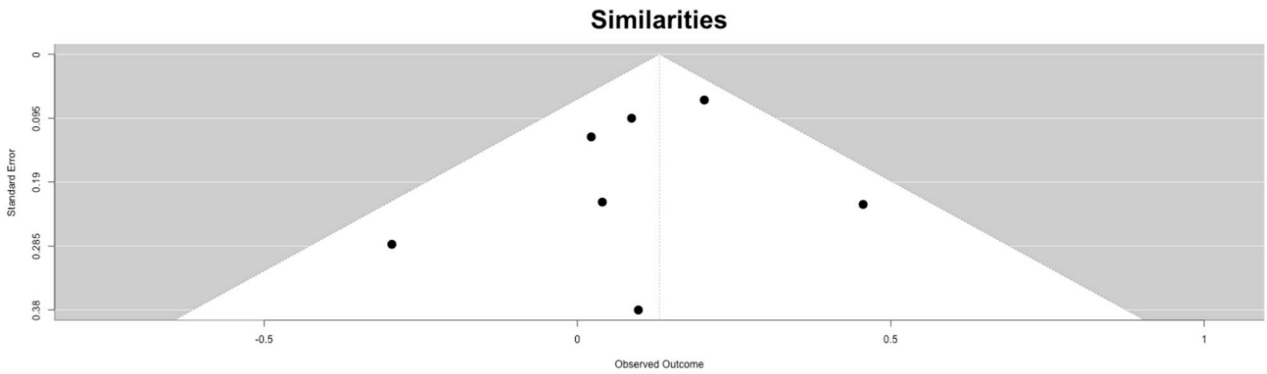

Figure S31. Funnel plot for the Coding subtest

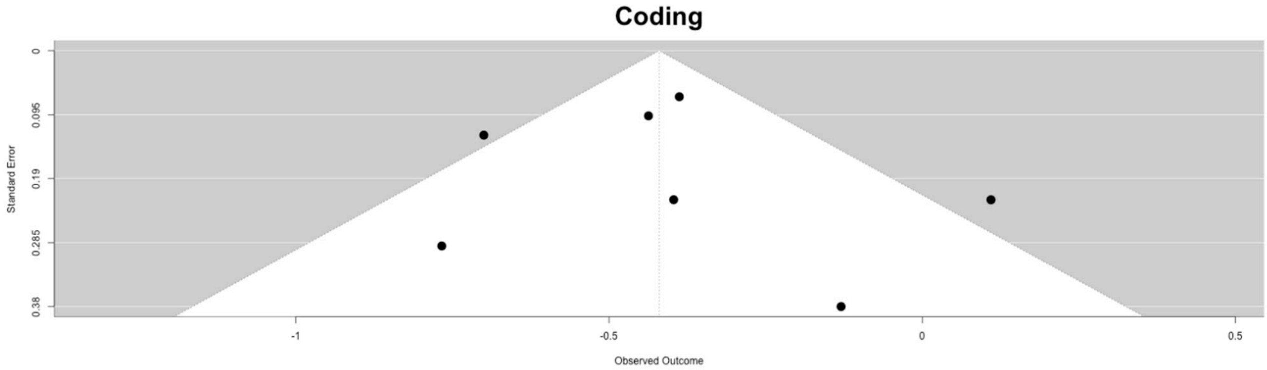

Figure S32. Funnel plot for the Object Assembly subtest

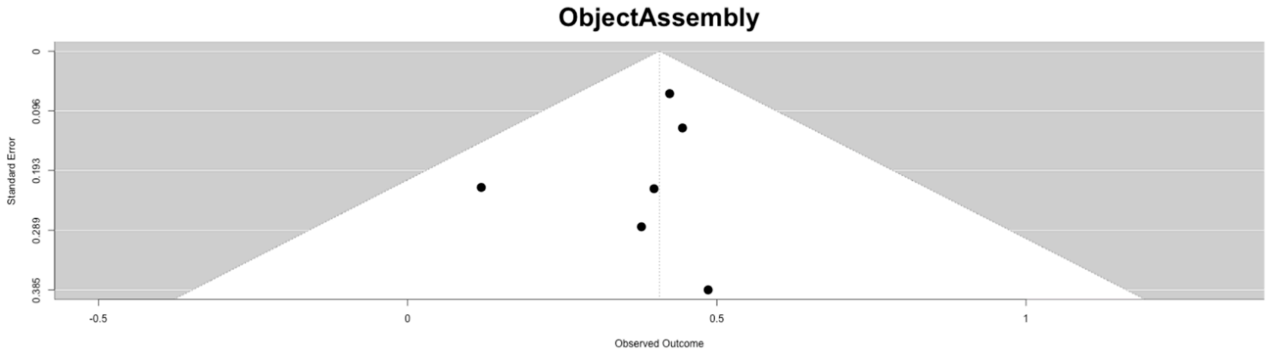

Figure S33. Funnel plot for the Picture Arrangement subtest

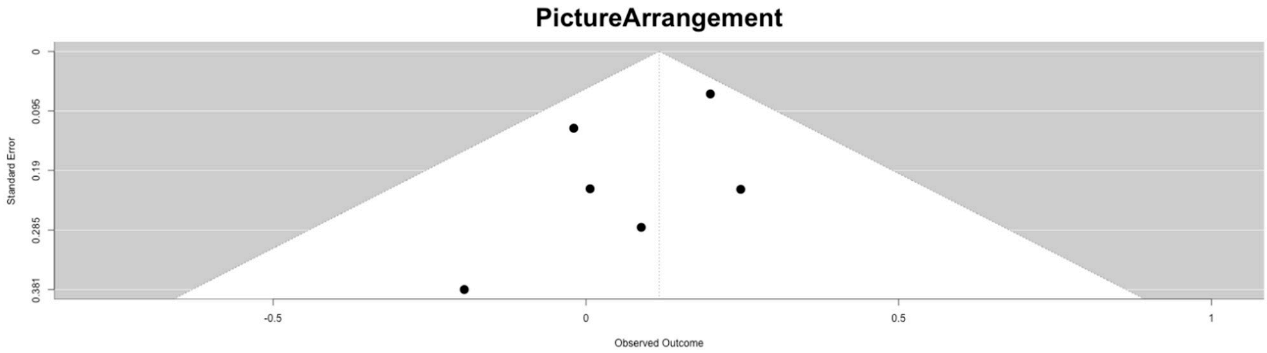

Figure S34. Funnel plot for the Picture Completion subtest

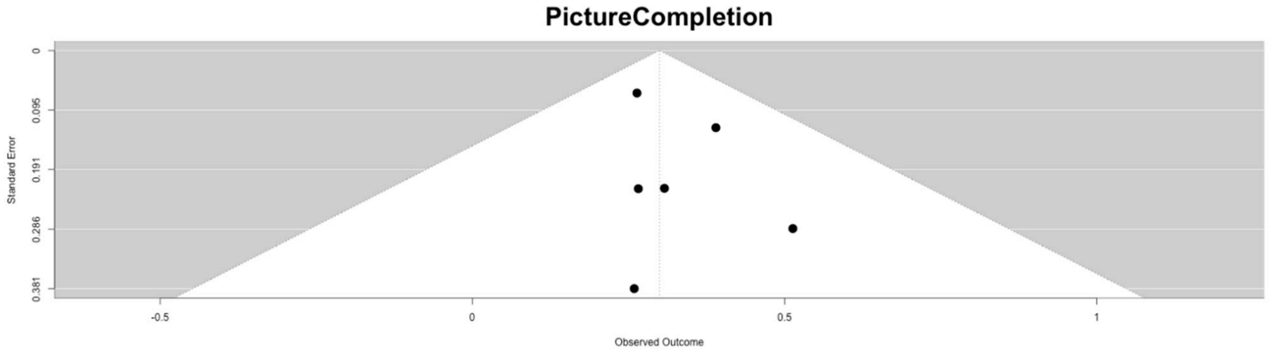

Figure S35. Funnel plot for the Information subtest

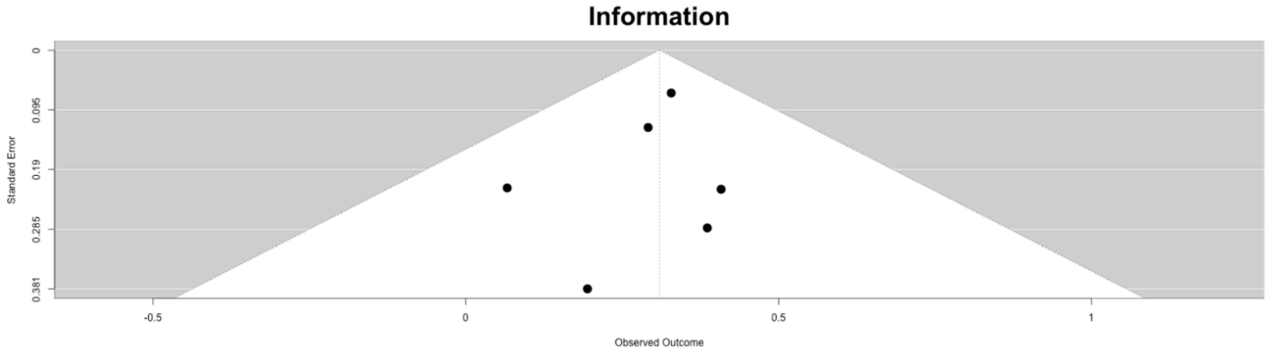

Figure S36. Funnel plot for the Arithmetic subtest

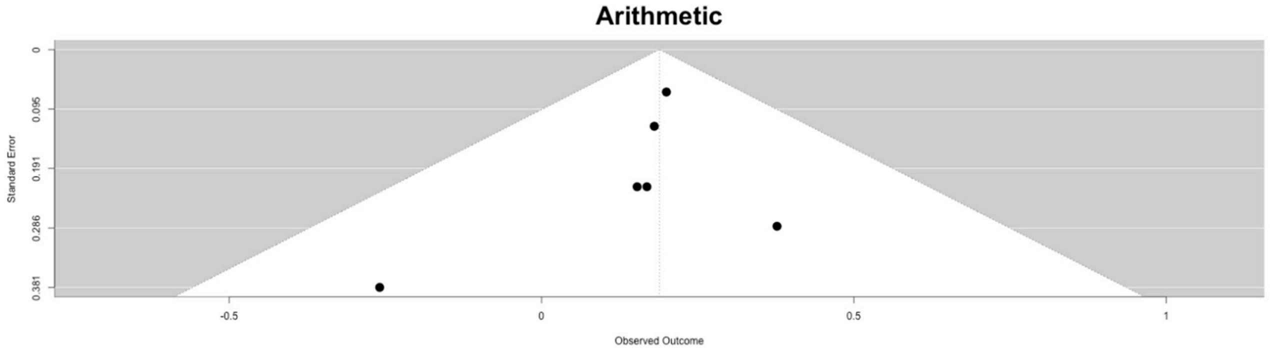

Supplement: Supplementary file 1 [file jintelligence-13-00018-s001.zip › jintelligence-3405711-supplementary.pdf]
